# Overcoming the Silencing of Doxycycline-Inducible Promoters in hiPSC-derived Cardiomyocytes

**DOI:** 10.12688/openreseurope.19024.1

**Published:** 2024-12-18

**Authors:** Michelle Guichardaz, Sveva Bottini, Elisa Balmas, Alessandro Bertero

**Affiliations:** 1 Department of Molecular Biotechnology and Health Sciences, Molecular Biotechnology Center “Guido Tarone”, University of Turin, Torino, 10126, Italy

**Keywords:** Human induced pluripotent stem cells, cardiomyocytes, inducible expression, silencing, TRE3VG promoter, T11 promoter, ubiquitous chromatin opening element, genomic safe harbours, sodium butyrate, Tet-On 3G

## Abstract

**Background:**

Human induced pluripotent stem cells (hiPSCs) are pivotal for studying human development, modeling diseases, and advancing regenerative medicine. Effective control of transgene expression is crucial to achieve temporal and quantitative precision in all of these contexts. The doxycycline (dox)-inducible OPTi-OX system, which integrates the Tet-On 3G transactivator and dox-responsive transgene at the *hROSA26* and *AAVS1* genomic safe harbors (GSHs), respectively, offers a promising solution. Yet, transgene silencing, particularly in hiPSC-derived cardiomyocytes (hiPSC-CMs), limits its utility.

**Methods:**

To address this, we evaluated strategies to enhance dox-inducible transgene expression. We compared two promoters, TRE3VG and T11, for activity and stability, and investigated the addition of a Ubiquitous Chromatin Opening Element (UCOE) to reduce silencing. We also tested relocating the transgene cassette to the *CLYBL* GSH, and employed sodium butyrate (SB), a histone deacetylase inhibitor, to restore promoter activity. Transgene expression was assessed *via* flow cytometry and real-time quantitative PCR.

**Results:**

TRE3VG exhibited higher activity than T11, but both were prone to silencing. UCOE did not enhance promoter activity in hiPSCs, but modestly reduced silencing in hiPSC-CMs. Targeting the *CLYBL* locus improved promoter activity compared to *AAVS1* in both hiPSCs and hiPSC-CMs. SB restored activity in silenced inducible promoters within hiPSC-CMs, but compromised hiPSC viability. Unexpectedly, Tet-On 3G was silenced in some clones and could not be reactivated by SB.

**Conclusions:**

These findings underscore the need for integrating multiple strategies, including careful GSH selection, improved cassette design, epigenetic modulation, and clone screening, to develop robust dox-inducible systems that retain functionality during hiPSC differentiation.

## Plain language summary

Human induced pluripotent stem cells (hiPSCs) have great potential for studying human development, modelling diseases, and developing cell and gene therapies. A significant challenge in using hiPSCs is controlling when and how much specific genes are expressed. This control is crucial for managing gene activity at different stages. However, the systems designed to induce gene expression often fail when the cells differentiate into specialized types, like heart cells. In this research project, we explored strategies to improve gene expression control in both hiPSCs and hiPSC-derived heart cells using a platform for optimized inducible gene overexpression (OPTi-OX). This approach relies on precise genome modifications of specific regions called genomic safe harbours (GSH). We tested two DNA fragments to drive inducible gene expression (promoters: TRE3VG and T11), and found that the TRE3VG promoter was more active. To enhance this further, we added another DNA fragment (Ubiquitous Chromatin Opening Element, or UCOE) before the TRE3VG promoter, which helped maintain gene activity during heart cell differentiation. We also inserted the TRE3VG promoter into a different GSH called *CLYBL*, which showed better results than a commonly used region known as *AAVS1*. While some silencing occurred during differentiation, the *CLYBL* region remained more active. Additionally, we tested a drug acting on the epigenome called sodium butyrate (SB), which helped to reverse silencing and boost gene expression, but reduced the viability of the hiPSCs. Overall, this research highlights the challenges of maintaining stable transgene expression during stem cell differentiation and offers insights into how we can control gene activity for research and medical applications.

## Introduction

Human pluripotent stem cells (hPSCs), including human embryonic stem cells (hESCs) and human induced pluripotent stem cells (hiPSCs), provide great possibilities for studying human development, modelling human diseases, and developing innovative cell treatments to treat pathological conditions. Achieving precise control over transgene expression in these cells and their differentiated derivatives is crucial ^1^. Strategies like cell and gene therapy, the transplantation of cells that deliver a gene cargo, and "forward programming", hPSCs differentiation into specific cell types through transcription factor overexpression, both rely heavily on the ability to fine-tune transgene activation ^1, 2^. Effective control of transgene activity is essential to minimize potential side or off-target effects and to improve the safety of gene therapy approaches ^3^.

The Tet-On system has emerged as a key inducible method for regulating transgene expression with temporal flexibility and high precision. It utilizes a reverse tetracycline-controlled transactivator (rtTA) and a doxycycline (dox)-responsive Tet promoter (TRE) to precisely control transgene induction ^4^. To optimize expression in hPSCs, some of us previously developed the optimized inducible overexpression (OPTi-OX) platform ^5^. This method relies on precise genome editing of the human *ROSA26* ( *hROSA26)* and *AAVS1* genomic safe harbours (GSH)—genomic regions that remain active across nearly all human cell types, resist transgene silencing, and can be modified without compromising cellular functionality ^6^—for stable expression of the third generation rtTA (Tet-On 3G) and the dox-inducible cassette driven by the TRE3VG promoter, respectively. This setup minimizes promoter interference and maximizes inducible transgene cargo capacity and copy number, overall enabling consistent, high-level expression in hPSCs ^5^.

However, a major limitation of TRE-based systems like OPTi-OX is promoter silencing across differentiation, which diminishes transgene expression in specific lineages, such as hPSC-derived cardiomyocytes (hPSC-CMs), smooth muscle cells, and hepatocytes ^7– 9^. This silencing may results from epigenetic modifications of the synthetic cassette, including DNA methylation and histone deacetylation, which negatively regulate transcription ^10– 12^. Additionally, the surrounding genomic environment and transcription factor accessibility can lead to transgene silencing.

We set out to identify methods to counteract silencing in both hiPSCs and hiPSC-CMs through promoter modification, chromatin environment modulation, and the use of alternative GSHs. First, we compared two dox-inducible promoters, TRE3VG (T3) and its modified variant, T11. TRE3VG derives from the Ptet-T1 promoter, which includes a minimal CMV promoter with randomized spacer sequences of 36 nucleotides between the seven tet operators (TetOs). Compared to this, TRE3VG presents point mutations establishing consensus sequences for the TATA-box and TFIIB binding site ^13^, enhancing transcription initiation and reducing background activity ^14– 18^. It also carries a deletion of the CMV 5'-untranslated region (5'-UTR), creating a truncated downstream promoter element (DPE) ^19^. The T11 promoter, in contrast, incorporates structural modifications such as the mouse mammary tumor virus (MMTV) promoter, Oct-1 binding sites, and a 5' untranslated region (5'-UTR) from the turnip yellow mosaic virus (TYMV) downstream of the initiator element ^13^. These changes may interact differently with silencing mechanisms and thus potentially enhance promoter stability.

Another strategy that we explored to counteract promoter silencing is integrating a Ubiquitous Chromatin Opening Element (UCOE) sequence upstream of the promoter. UCOEs, characterized by unmethylated CpG islands that include bidirectional promoters associated with housekeeping genes, establish open chromatin environments that resist epigenetic silencing across various cell types, including iPSCs and their derivatives ^20, 21^. We employed a minimal 0.7 kb version of the A2UCOE, lacking the HNRPA2B1 promoter, to maintain an open chromatin state without introducing aberrant splicing events associated with larger UCOE constructs ^20, 22^.

We also tested relocating the dox-inducible cassette from the *AAVS1* GSH on chromosome 19 to the *CLYBL* locus on chromosome 13. While *AAVS1*, presenting native insulators, has been widely used for its open chromatin structure that favours transcriptionally productive integration, the *CLYBL* locus has been demonstrated to support more robust expression in hPSCs and their derivatives ^1, 7, 23– 25^. Additionally, homozygous loss-of-function variants in *CLYBL* are merely linked to reduced vitamin B12 levels in otherwise healthy individuals, demonstrating that *CLYBL* is not an essential gene for life and is therefore "safe" for clinical use ^26^.

Lastly, we explored whether histone deacetylase (HDAC) inhibition using sodium butyrate (SB) can restore promoter activity in silenced hiPSCs and hiPSC-CMs. Studies suggest that SB effectively reverses silencing by enhancing histone acetylation, thereby reactivating transgene expression without impairing cell viability or proliferation ^27^.

In all, understanding how these strategies influence transgene regulation will inform future efforts to optimize inducible systems for regenerative medicine and disease modelling.

## Methods

### hiPSCs culture

Healthy male hiPSCs with a TTN-GFP knock-in reporter (Allen Institute for Cell Science, #AICS-0048-039) were maintained under feeder-free conditions at 37 °C, 5% CO2, and normoxia. Cells were seeded onto culture dishes coated with LDEV-Free Geltrex (Gibco #A1413302) [diluted in DMEM-F12 (Gibco #31330038) to a final concentration of 17 μg mL ^-1^], and cultured in Essential 8 medium (Gibco #A1517001). Colonies were split into small clumps every 4–5 days using Versene [0.5 mM EDTA (Sigma-Aldrich #4055) diluted in PBS (Euroclone #ECB4053)] and passaged in media supplemented with 2 μM Thiazovivin (Cayman Chemicals #004CA14245-25). The medium was changed daily.

### Plasmids

Plasmid assembly was carried out using the NEBuilder HiFi DNA Assembly Master Mix (NEB #BE5520S), while ligation was performed with T4 DNA Ligase (ThermoFisher #EL0012). Vector digestion was performed with restriction enzymes (NEB/ThermoFisher), and PCR amplification was conducted using Q5 Hot Start High-Fidelity DNA Polymerase (NEB #BM0493L) and primers obtained from Eurofins Genomics. Before assembly, cut vectors were dephosphorylated using Fast Alkaline Phosphatase (ThermoFisher #EF0651). DNA extraction from agarose gels was done using the QIAEX II Gel Extraction Kit (QIAGEN #20021), and PCR products were purified with the Nucleospin Gel and PCR Clean-up kit (MACHERY-NAGEL #740609.50). Recombinant plasmids were transformed into NEB 5-alpha Competent *E. coli* (High Efficiency, NEB #C2987). Plasmid preparations were performed with the Monarch Plasmid Miniprep Kit (NEB #T1110) and the QIAfilter Plasmid Midi Kit (QIAGEN #12243), using QIAGEN EndoFree Plasmid Buffer Set (#19048) for plasmids intended for genome editing. All procedures followed the manufacturer’s protocols. Additional molecular biology techniques, such as electrophoresis and *E. coli* culture, adhered to standard protocols. All plasmids were fully sequence-verified by Plasmidsaurus (Eugene, OR, USA).

pR26-Bst_CAG-Tet-On-3G was generated starting by replacing the EGFPd2 cDNA of pR26-Bst_CAG-EGFPd2 ^7^ with a synthetic Tet-On 3G cDNA fragment (Clonetech) using MluI and BamHI.

pAAV-puro_TRE3VG_EGFP-Responder was synthetized based on the previously published sequence of pAAV-Puro_TRE-EGFP ^5^.

pAAV-puro_T11_EGFP-Responder was constructed by excising TRE3VG using SphI and SpeI restriction enzymes from pAAV-puro_TRE3VG_EGFP-Responder and replacing it through a NEBuilder Hi-Fi reactions with PCR amplified TRE3VG fragment from pAAV-puro_TRE3VG_EGFP-Responder with primers 5’-GAAGACAATAGCAGGCATG–3’ and 5’-CCTGGTTTACATAAGCACTCGACATACGTTCTC-3’, and PCR amplified T11 sequence from pRRL-TRE3GS(T11) (a kind gift of Johannes Zuber) with primers 5’-TGCTTATGTAAACCAGGGC-3’ and 5’-CTCACCATGGTGGCACTAGTTTACGAGGGTAGGAAGTG-3’.

pAAV-puro_TRE3VG_mCherry-Responder was generated by excising EGFP using NcoI and EcoRI restriction enzymes from pAAV-puro_TRE3VG_EGFP-Responder and replacing it through NEBuilder Hi-Fi with mCherry sequence PCR amplified from pHEC015_ *CLYBL*_neo_CAG_dCas9-P2A-T2A-mCherry (a kind gift of Jesse Zalatan) with primers 5’-CTTATACTTACTAGTGCCACCATGGTGAGCAAGGGCG–3’ and 5’-CGGGTACCGAGCTCGAATTCTTACTTGTACAGCTCGTCCATGCCG-3’.

pAAV-puro_UCOE_TRE3VG_mCherry-Responder was constructed by linearizing the vector pAAV-puro_TRE3VG_mCherry-Responder using PspXI followed by the addition through NEBuilder Hi-Fi of the UCOE sequence PCR amplified from *CLYBL* hOPM (Addgene #112499) using primers 5’-GGTGGGCTCTATGGTCACTCGAGTACGGGAGGTGGTCCCTGCAG-3’ and 5’-CACTGATAGGGAGTAAACTCGAGTCCTCCGCGCCTACAGCTCAAGCCAC-3’.

pCLYBL-Neo_TRE3VG_mCherry_Responder was created by excising the cassette encoding for dCAS9 and mCherry using AscI and KpnI restriction enzymes from pHEC015_ *CLYBL*_neo_CAG_dCas9-P2A-T2A-mCherry and replacing it through NEBuilder Hi-Fi with TRE3VG-mCherry cassette PCR amplified from pAAV-puro_TRE3VG_mCherry-Responder using primers 5’-CAGTGAGAATATTGG-CGCGCCGGTGGGCTCTATGGTCACTC-3’ and 5’-GTTAACTTAATTAAGGTACCCGACTTAAGAGTACTGTCAAGGCAGTG-3’.

### Plasmid delivery

For lipofection, hiPSCs were washed with DPBS and dissociated into single cells using Versene for 3 minutes at 37 °C. Then, 90% of Versene was removed and cells were incubated for other 2 minutes at 37 °C. A total of 200,000 cells were resuspended in media supplemented with 2 μM Thiazovivin and seeded into a Geltrex-coated well of a 6-well plate. After 24 h, the media was replaced with Essential 8 (Gibco #A1517001), supplemented with a transfection mix composed of 200 μl Opti-MEM Reduced Serum Medium (Gibco #31985047), 2 μl Lipofectamine Stem Transfection Reagent (Invitrogen #STEM00001), and 2 μg total DNA. After 4 h, the media was replaced with fresh Essential 8.

For nucleofection, hiPSCs were washed with DPBS and dissociated into single cells using StemPro Accutase (Gibco # A1110501) for 5 minutes at 37 °C. A total of 1 million cells were resuspended in 100 μl P3 Primary Cell 4D-Nucleofector solution (Lonza #v4XP-3024) and transferred to the conductive polymer nucleovette. Each condition used 10 μg of DNA. The nucleovette was then loaded into the Lonza 4D-Nucleofector System X Unit and subjected to the CA137 program. Nucleofected cells were seeded into a Geltrex-coated well of a 24-well culture dish using recovery media supplemented with the CEPT cocktail [50 nM Chroman1 (MedChem Express #HY-15392), 5 μM Emricasan (Selleckchem #S7775), Polyamine supplement 1:1000 (Sigma-Aldrich #P8483), 0.7 μM Trans-ISRIB (Tocris #5284)]. The following day, the media was refreshed to remove the CEPT. Cells were passaged into a 10 cm culture dish 48h post-nucleofection.

### Genome editing

Genome editing at the *hROSA26* locus was performed with a pair of plasmids encoding for sgRNA and Cas9 D10 nickase mutant, as previously described ^7^. hiPSCs were co-transfected with pSpCas9n(BB)_R26-L, pSpCas9n(BB)_R26-R, and pR26-Bst_CAG-Tet-On-3G (at a 1:1:2 ratio). Starting 48 post-transfection, cells were selected with by adding 4 µL mL ^-1 ^Blasticidin S (Sigma-Aldrich # SBR00022) for 5 days, with 2 μM Thiazovivin added on days 2 and 3 to enhance single-cell survival.

Genome editing at the *AAVS1* locus was carried out using obligate heterodimer zinc finger nucleases (ZFNs) , as previously described ^7^. hiPSCs were co-transfected with pZFN- *AAVS1*_ELD (Addgene #159297), pZFN- *AAVS1*_KKR (Addgene #159298), and a pAAV targeting plasmid among those described in the previous section (at a 1:1:2 ratio). Starting 48 h post-transfection, cells were selected with 0.5 μg mL ^-1 ^Puromycin (Gibco #A1113803) for 5 days, with 2 μM Thiazovivin added on days 2 and 3 to enhance single-cell survival.

Genome editing at the *CLYBL* locus was executed through transcription activator-like effector nucleases (TALENs). hiPSCs were co-nucleofected with pZT-C13-L1 (Addgene #62196), pZT-C13-R1 (Addgene #62197), and p *CLYBL*-Neo_TRE3VG_mCherry_Responder (at a 1:1:2 ratio). Genome edited cells were selected 24 h after seeding into 10 cm culture dishes, using 50 μg mL ^-1^ Geneticin (Gibco #10131035) for 7 days, with 2 μM Thiazovivin added on days 2 and 3 to enhance single-cell survival.

### Genotyping

hiPSC clones generated from genome editing experiments were manually picked, expanded, and subjected to genomic PCR screening to confirm site-specific, on-target integration of the transgene and to check for any random integration of the targeting plasmid elsewhere in the genome. To assess site-specific transgene integration (INT), 5’INT and 3’INT junctional PCRs were conducted using a primer positioned at either the 5’ or 3’ end of the transgene and a second primer located on the genomic locus outside the relevant homology arm. Wild-type (WT) PCR was performed using two primers that map to the genomic locus outside the homology arms to determine whether one or both alleles had been edited. To detect any potential off-target random integration, two additional backbone (BB) PCRs were conducted: 5’BB and 3’BB PCRs, each using one primer specific to either the 5’ or 3’ end of the transgene and a second primer targeting the plasmid backbone outside the homology arms. PCR reactions were carried out according to the following protocol. Genomic DNA (10 ng) was amplified in a reaction mixture containing 1× LongAmp Taq Reaction Buffer (NEB #B0323S), 300 µM dNTP mix (NEB # BN0447L), 250 nM forward primer, 250 nM reverse primer, 2% DMSO >-99.7% (Fisher scientific #BP231-100), and 2 U of LongAmp Taq DNA Polymerase (NEB #M0323L). The reaction volume was adjusted to 10 µL with nuclease-free water (Qiagen # 129115). All primer combinations used in these assays and PCR conditions are listed in Table 1. Table 2 summarizes all the gene targeting experiments and their efficiency.

**Table 1.** Genotyping strategies.

GSH	PCR type	Dir.	Primer sequence 5’ – 3’	Amplicon wild type (bp)	Amplicon transgene (bp)	Amplicon plasmid (bp) ^a^	T ann. ^b^ (°C)	Ext. time ^b^
*hROSA26*	LOCUS	FW	GAGAAGAGGCTGTGCTTCGG	2186	Variable ^c^ (LoA for CAG)	No band	63	>3’ ^c^
		REV	ACAGTACAAGCCAGTAATGGAG					
	5’ INT	FW	GAGAAGAGGCTGTGCTTCGG	No band	1274	No band	60	1’ 30’’
		REV	AAGACCGCGAAGAGTTTGTCC					
	3’ INT	FW	GAGAATAGCAGGCATGCTG	No band	1020	No band	60	2’
		REV	ACAGTACAAGCCAGTAATGGAG					
	5’ BB	FW	CGTTGTAAAACGACGGCCAG	No band	No band	1035	60	1’ 30’’
		REV	AAGACCGCGAAGAGTTTGTCC					
	3’ BB	FW	GAGAATAGCAGGCATGCTG	No band	No band	919	60	1’ 30’’
		REV	TGACCATGATTACGCCAAGC					
*AAVS1*	LOCUS	FW	CTGTTTCCCCTTCCCAGGCAGGTCC	1692	Variable ^c^	No band	65	>3’ ^c^
		REV	TGCAGGGGAACGGGGCTCAGTCTGA					
	5’ INT	FW	CTGTTTCCCCTTCCCAGGCAGGTCC	No band	1113	No band	72 to 64	1’ 30’’
		REV	TGAGGAAGAGTTCTTGCAGCTC					
	3’ INT	FW	GAATTCGAGCTCGGTACC	No band	1166	No band	60	1’ 30’’
		REV	TGCAGGGGAACGGGGCTCAGTCTGA					
	5’ BB	FW	GCTTGTCTGTAAGCGGATGC	No band	No band	1424	60	1’30’’
		REV	TGAGGAAGAGTTCTTGCAGCTC					
	3’ BB	FW	GAATTCGAGCTCGGTACC	No band	No band	1227	60	1’30’’
		REV	ATGCTTCCGGCTCGTATGTT					
*CLYBL-*	LOCUS	FW	TAAGTGACCCCTGGCGAGAC	1728	Variable ^c^	No band	65	>3’ ^c^
		REV	AGATGGAGCAGTGGATGACA					
	5’ INT	FW	TAAGTGACCCCTGGCGAGAC	No band	962	No band	65	1’ 30’’
		REV	AAGACTTCCTCTGCCCTCTC					
	3’ INT	FW	TCCAGGACGGAGTCAGTGAG	No band	907	No band	65	1’ 30’’
		REV	AGATGGAGCAGTGGATGACA					
	5’ BB	FW	CACAGGAAACAGCTATGACC	No band	No band	980	65	1’ 30’’
		REV	AAGACTTCCTCTGCCCTCTC					
	3’ BB	FW	TCCAGGACGGAGTCAGTGAG	No band	No band	874	65	1’ 30’’
		REV	TTTTCCCAGTCACGACGTTG					

^a^ Result of PCR on targeting vector (positive control for off-target plasmid integration). ^b^ Variable parameters in PCR program: (1) 94 °C 5’; (2) 94 °C 15”; (3) T ann. 30”; (4) 65 °C Ext. time; (5) Repeat 2-4 for 34 cycles; (6) 65 °C 5’; (7) 10 °C hold. T ann. was decreased by 1 °C per cycle during cycles 2-9 of the *AAVS1 5’* INT PCR. ^C^ Size dependent on the transgene size; Ext. time set accordingly.

**Table 2.** Genotyping results.

Donor plasmid	Clones	Fail ^a^	Rand. Int. ^b^	Het + extra ^c^	Hom + extra ^c^	Het	Hom	Correct (%) ^d^	Total (%) ^e^
pAAV-puro_TRE3VG_EGFP-Responder	3	0	0	0	1	1	1	66	100
pAAV-puro_T11_EGFP-Responder	3	0	0	1	1	1	0	33	100
pAAV-puro_TRE3VG_mCherry-Responder	14	0	0	5	7	0	2	14	100
pAAV-puro_UCOE_TRE3VG_mCherry-Responder	23	1	0	11	3	5	4	39	96
pCLYBL-Neo_TRE3VG_mCherry_Responder	5	0	0	5	0	0	0	0	100

^a^ Evidence of targeting, but incorrect size of 5’ or 3’ INT PCR ^b^ No evidence of targeting (lack of bands in 5’ and 3’ INT PCR, and presence of WT band in locus PCR) ^c^ Correct targeting, but with additional random integration of the targeting plasmid (bands in 5’ and/or 3’ BB PCR ^d^ Heterozygous (het) and homozygous (hom) targeting ^e^ Including clones with additional random integration of the targeting plasmid (het + extra and hom + extra)

### Induction of reporter transgene expression

Doxycycline hyclate (Sigma-Aldrich #D9891-1G) was added to the culture media at 1 μg mL ^-1^ to induce reporter transgene expression. The duration of the treatment is described for each experiment in the results and/or the figure legends. During this treatment media was changed every day.

### Imaging of reporter transgene expression

Fluorescence microscopy images were acquired with an Axiovert 5 fluorescence microscope (Zeiss) equipped with Colibri 3 LED modules for 469/38 nm and 555/30 nm excitation.

### Reverting promoter silencing

Sodium Butyrate (Sigma-Aldrich #303410-100G) was added to the culture media at 500 uM in combination with doxycycline hyclate. The duration of the treatment is described for each experiment in the results and/or the figure legends. During this treatment media was changed every day.

### Flow cytometry

The expression of fluorescent reporters was assessed in live cells. hiPSCs were harvested by incubating them at 37 °C for 3 minutes in a solution containing TrypLE 1× (Gibco #12605010) and Versene at a 3:1 ratio. CMs were harvested using a 2.5% Trypsin solution (Euroclone #ECB3051D) diluted 1:10 in Versene, followed by incubation at 37 °C for 5 minutes. Post-harvest, cells were centrifuged at 100 g for 5 minutes for hiPSCs and 190 g for 5 minutes for CMs, then resuspended in FACS buffer [PBS with 2% FBS (LifeTechnologies #10270106) and 10 mM HEPES (FisherBioreagents #10756254)]. To label dead cells, Fixable Viability Dye eFluor 780 (Invitrogen #65-0865-14) was added to the sorting buffer. Live cells were kept on ice for analysis using a BD FACSVerse Cell Analyzer (EGFP expression) or a Sony SH800S cell sorter (mCherry expression). Data analysis was subsequently performed using FlowJo (v10). For analyses of median fluorescence intensity (MFI), values were normalized by dividing the MFI of treated samples per the MFI of the negative control not expressing the fluorophore.

### Cardiomyocyte differentiation

hiPSCs were washed once with DPBS and detached by incubating with Versene for 5 minutes. These cells were resuspended in Essential 8 media supplemented with 2 μM Thiazovivin and seeded into Geltrex-coated wells of a 12-well plate. Two different seeding densities were tested: 66 cells cm ^-2^ and 90 cells cm ^-2^. The next day, hiPSCs were primed in Essential 8 media supplemented with 1 μM CHIR99021 (Cayman chemicals #004CA13122-10) for 24 h. On day 0 of differentiation, mesoderm induction was initiated using CHIR99021 (5 μM) in RBA media [RPMI 1640 (Gibco #61870010) supplemented with 500 μg mL ^-1^ Bovine Serum Albumin (Sigma-Aldrich #A8412-100ML) and 213 μg mL ^-1^ Ascorbic Acid (Sigma-Aldrich #49752-10G)]. After 48 h, on day 2 of differentiation, cells were washed with DPBS, and cardiac progenitor induction was carried out with 2 μM Wnt-C59 (Cayman chemicals #004CA16644-10) in RBA media. After another 48 h, on day 4, cells were washed again with DPBS and incubated in plain RBA media for an additional 48 h. From day 6 until the analysis CMs were cultured in RPMI-B27 media [RPMI 1640 supplemented with B-27 (Gibco #17504044)], with media changes every 48 h.

### RT-qPCR

RNA was extracted using the Quick-RNA ^TM^ MiniPrep Kit (Zymo Research #ZYR1055). cDNA synthesis was then carried out with random primers using 500 ng of total RNA and the High Capacity cDNA Reverse Transcription Kit (Applied Biosystems #4368813). qPCR reactions were set up in 10 μL volumes containing 10 ng cDNA, 250 nM of each PCR primer, and 1X Luna Universal qPCR MasterMix (NEB #BM3003E). Real-time quantitative PCR (RT-qPCR) was conducted in technical duplicates using a QuantStudio 6 Flex Real-Time PCR System (Applied Biosystems). The qPCR primer sequences are listed in Table 3. Transgene expression levels were normalized to one of two housekeeping genes: *HPRT1* for hiPSCs and *PBGD* for hiPSC-derived CMs.

**Table 3.** RT-qPCR primers.

Gene	Orientation	Primer sequence 5’ – 3’
*HPRT1*	FW	TGACACTGGCAAAACAATGCA
	REV	GGTCCTTTTCACCAGCAAGCT
*PBGD*	FW	GGAGCCATGTCTGGTAACGG
	REV	CCACGCGAATCACTCTCATCT
Tet-On 3G	FW	GAAGGCCTGACGACAAGGAA
	REV	GTCGCGATGTGAGAGGAGAG

### Isolation and sequencing of Tet-On 3G

hiPSCs clones silenced for Tet-On 3G were analysed through 3’-INT genomic PCR to confirm the integration of the transactivator at the *hROSA26* GSH. PCR reaction was carried out using LongAmp Taq DNA Polymerase following the previously described protocol (genotyping) and using the primers 5’-GAAGGCCTGACGACAAGGAA-3’ (FW) and 5’-ACAGTACAAGCCAGTAATGGAG-3’ (REV). PCR products were purified with the Nucleospin Gel and PCR Clean-up kit (MACHERY-NAGEL #740609.50) and sequenced using the Mix2Seq kit (Eurofins genomics).

### Statistical analysis

Statistical analysis was performed with GraphPad Prism (v10.3.1). Data is presented as mean ± SEM. The number of replicates and the statistical test used are reported in the figure legends.

## Results

### Comparison of TRE3VG and T11 promoter activity in hiPSCs for inducible transgene expression

**Figure 1.**
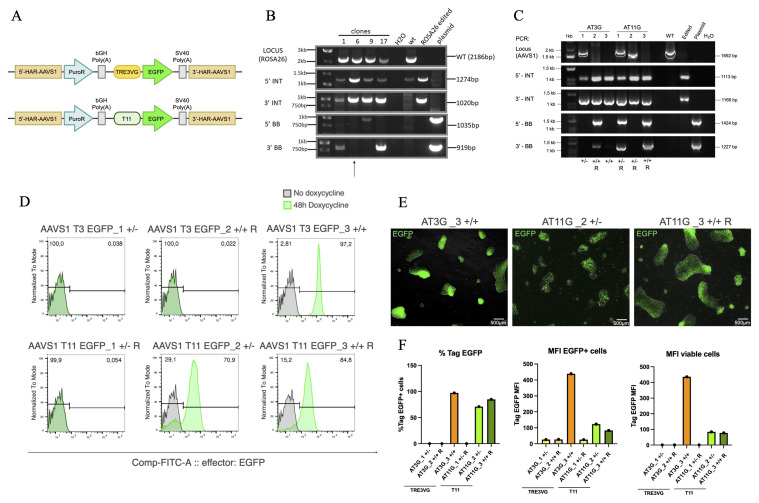
Comparison of TRE3VG and T11 promoter activity in hiPSCs for inducible transgene expression. ( **A**) Schematic of the genome editing strategy. pAAV-puro_TRE3VG_EGFP-Responder (top) and pAAV-puro_T11_EGFP_Responder (bottom) contain the TRE3VG and T11 promoters, respectively. Both plasmids target the *AAVS1* GSH and incorporate a puromycin resistance gene trap for selection. 5’/3’-HAR: upstream/downstream homology arm region; PuroR: puromycin-N-acetyltransferase; bGH Poly( **A**): bovine growth hormone polyadenylation site; SV40 Poly ( **A**): simian virus 40 polyadenylation site; TRE3VG/T11: doxycycline (dox)-responsive promoters; EGFP: enhanced green fluorescent protein. ( **B**) Genomic PCR confirming the integration of the Tet-On 3G transactivator into the *hROSA26* locus in WTC11 TTN-GFP hiPSCs. Black lines show the expected amplicon sizes ( Table 1). Clone 6 was selected for further experiments (henceforth WTC11 Tet-On 3G). WT: wild-type hiPSCs; ROSA26 edited: pool of hiPSCs edited at the *hROSA26* locus; plasmid: targeting plasmid; H2O: no template control; 1kb ladder; LOCUS: PCR of target region on *hROSA26* (results in loss of amplification for homozygous editing); 5’/3’ INT: PCR of transgene 5’/3’ end integration region (indicative of expected transgene targeting); 5’/3’ BB: PCR of vector backbone 5’/3’ end integration region (indicative of non-specific off-target plasmid integration). ( **C**) Genomic PCR verifying site-specific retargeting of the *AAVS1* locus in WTC11 Tet-On 3G hiPSCs. AT3G: pAAV-puro_TRE3VG_EGFP-Responder; AT11G: pAAV-puro_T11_EGFP_Responder. +/+:homozygous; +/-: heterozygous; R: random integration. Refer to panel B for other abbreviations. ( **D**– **E**) Flow cytometry analyses ( **D**) and fluorescence microscopy images ( **E**) of EGFP expression after treatment of hiPSCs clones with dox for 48 h. In E, scale bars: 500 μm ( **F**) Flow cytometry quantifications of data from panel D.

To start, we evaluated the activity of the TRE3VG and T11 promoters in hiPSCs. Our hypothesis was that the T11, being structurally different from the TRE3VG, could interact differently with the mechanisms leading to silencing, therefore preventing the inhibition of the transgene. We used two plasmids which express the TRE3VG and T11 promoters, respectively, upstream of the Enhanced Green Fluorescent Protein (EGFP) cDNA. Furthermore, they present short homology arms to the *AAVS1* GSH and a puromycin resistance gene trap ( Figure 1A). EGFP was chosen to facilitate the evaluation and comparison of promoter activity based on the percentage of EGFP+ cells and their Median Fluorescence Intensity (MFI). We co-transfected these plasmids with those encoding for ZFNs targeting the site of desired integration into WTC11 TTN-GFP hiPSCs that had been previously engineered in heterozygosity at the *hROSA26* locus to express a single copy of the Tet-On 3G transactivator (WTC11 Tet-On 3G cell line). Genomic PCR to confirm the integration of Tet-On 3G is reported in Figure 1B. This setup allowed us to test the OPTi-OX system. 24 h post-transfection, hiPSCs with integrated plasmids containing a puromycin resistance cassette were selected with puromycin. The surviving cells were then subjected to monoclonal isolation and expansion. Genomic PCR was used to verify site-specific targeting of the clonal lines ( Figure 1C). We isolated 6 clones which survived the antibiotic selection: 3 of these carried integration observed in one allele, while the other 3 were integrated in both alleles, with or without additional random integrations, overall with a correct targeting efficiency of 66%, as shown in Table 2

Next, the selected clones—three *AAVS1* T3 EGFP (AT3G_1/2/3) and three *AAVS1* T11 EGFP (AT11G_1/2/3)—were analysed by flow cytometry to compare EGFP expression after 48 h of dox treatment. Figure 1D reports that, among the TRE3VG promoter clones, two out of three did not express EGFP, while the AT3G_3 clone, carrying homozygous *AAVS1* transgene integration and no additional randomly integrated copies, exhibited homogeneous transgene expression (97.2% EGFP+). For the T11 promoter, one out of three clones was silenced, while the other two clones expressed EGFP, albeit with some heterogeneity (70.9% and 84.8% EGFP+). This variability is further illustrated by fluorescent microscopy images ( Figure 1E). To rigorously compare promoter activity, we assessed the MFI of all EGFP-expressing clones. Figure 1F demonstrates that the MFI was substantially (>4-fold) higher in the AT3G_3 clone expressing the TRE3VG promoter compared to the clones with the T11 promoter. A summary of genotype, percentage of EGFP positive cells, and MFI for all analysed clones is provided in Table 4.

**Table 4.** Flow cytometry result for genome edited hiPSC clones.

Clone ^a^	GSH genotype	Random integration	%GFP+/ mCherry+ ^b^	MFI GFP+/ mCherry+ ^c^
AT3G_1	+/-	No	0	26.23
AT3G_2	+/+	Yes	0	26.9
AT3G_3	+/+	No	97.2	439.67
AT11G_1	+/-	Yes	0	25.5
AT11G_2	+/-	No	70.9	123
AT11G_3	+/+	Yes	84.8	88.33
AT3C_1	+/+	Yes	99.5	413.46
AT3C_2	+/+	No	99.9	385.19
AT3C_3	+/+	Yes	0.10	5.74
AT3C_4	+/+	No	75.8	376.20
AT3C_5	+/+	Yes	85.5	405.17
AUT3C_1	+/+	No	99.1	358.85
AUT3C_2	+/+	No	99.9	516.55
AUT3C_3	+/+	No	0.39	5.4
AUT3C_4	+/+	Yes	97.0	419.07
AUT3C_5	+/+	Yes	99.1	454.41
AUT3C_6	+/+	Yes	69.8	565.80
CT3C_1	+/-	Yes	98.4	1156.67
CT3C_2	+/-	Yes	99.8	1192.32
CT3C_3	+/-	Yes	0.11	5.22
CT3C_4	+/-	Yes	91.6	1133.50
CT3C_5	+/-	Yes	79.3	390.42

^a^ AT3G: pAAV-puro_TRE3VG_EGFP-Responder; AT11G: pAAV- puro_T11_EGFP_Responder; AT3C: pAAV-puro_TRE3VG_mCherry-Responder; AT3UC: pAAV- puro_UCOE_TRE3VG_mCherry-Responder; CT3C: pCLYBL-Neo_TRE3VG_mCherry_Responder ^b^ Fluorophore dependent on targeting plasmid ^c^ Median fluorescence intensity of only fluorophore-positive cells, relative to fluorophore-negative control

Overall, these results indicated that, unexpectedly, the OPTi-OX system was not active in all hiPSC clones. Notably, our subsequent investigation into other completely silenced clones revealed that OPTi-OX inactivity was due to silencing of Tet-On 3G expression, not necessarily of the TRE3GV promoter itself—an aspect that will be explored further later in this manuscript. This experiment nonetheless allowed us to compare the two tested promoter, showing that TRE3VG promoter is >4-fold stronger compared to the T11 promoter. Based on these findings, we concluded that the TRE3VG promoter was more suitable for our study and selected it for further experimentation.

### Testing the Addition of UCOE to Enhance Inducible Transgene Expression in hiPSCs

**Figure 2.**
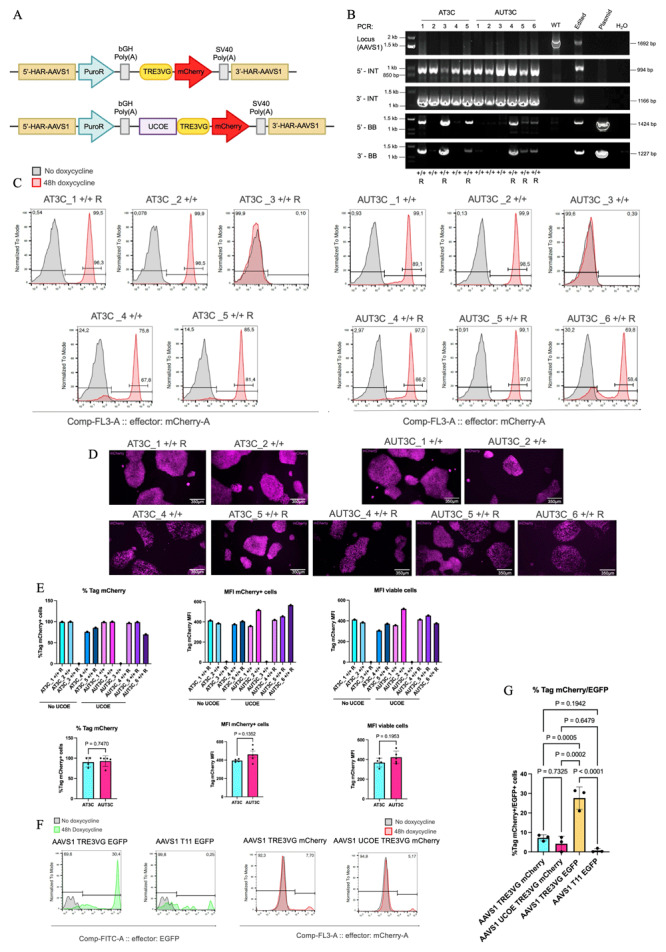
Testing the addition of UCOE to enhance inducible transgene expression in hiPSCs. ( **A**) Schematic of the genome editing strategy. pAAV-puro_TRE3VG_mCherry-Responder (top) and pAAV-puro_UCOE_TRE3VG_mCherry-Responder (bottom) contain the TRE3VG alone or preceded by the UCOE, respectively. Both plasmids target the *AAVS1* genomic locus and incorporate a puromycin resistance gene trap for selection. UCOE: ubiquitous chromatin opening element. Refer to Figure 1A for other abbreviations. ( **B**) Genomic PCR verifying site-specific targeting of the *AAVS1* locus in WTC11 Tet-On 3G hiPSCs. AT3C: pAAV-puro_TRE3VG_mCherry-Responder; AUT3C: pAAV-puro_UCOE_TRE3VG_mCherry-Responder. Refer to Figure 1B for other abbreviations. ( **C**– **D**) Flow cytometry analyses (C) and fluorescence microscopy images (D) of mCherry expression after treatment of hiPSC clones with dox for 48 h. In D, mCherry signal is shown in magenta pseudocolour to enhance visibility for colourblind individuals; scale bars: 350 μm ( **E**) Flow cytometry quantifications in individual clones from panel C (top), and aggregate data for all clones from the same experimental condition (bottom). Statistical analyses by two-tailed t-test (n = 4–5 clones). AT3C_1, AT3C_2, AUT3C_1, and AUT3C_2 clones were selected to proceed with cardiac differentiations. ( **F**) Representative flow cytometry analyses of mCherry and EGFP expression in WTC11 Tet-On 3G hiPSCs retargeted in pool with the *AAVS1* targeting plasmids described in Figure 1A and panel A. Cells were treated with dox for 48 h to induce transgene expression. ( **G**) Flow cytometry quantifications of three independent replicates of the experiment described in ( **F**). Statistical analysis by one-way ANOVA followed by Tukey’s post hoc multiple comparison test.

After identifying TRE3VG as a suitable promoter, we compared its activity alone and with a minimal UCOE sequence in hiPSCs and hiPSC-CMs. Our hypothesis was that the UCOE, being linked to an open chromatin environment, might reduce the silencing of TRE3VG. The WTC11 Tet-On 3G cell line had been derived in hiPSCs that express EGFP when the *TTN* gene, a marker of CMs, is active; thus, to avoid confusion with the fluorescent reporter for inducible promoter activity, we replaced EGFP with mCherry. We then cloned the UCOE sequence upstream of the TRE3VG promoter, resulting in two *AAVS1* targeting plasmids containing the TRE3VG promoter and mCherry without or with UCOE, respectively ( Figure 2A). We repeated the procedure to integrate these cassettes at the *AAVS1* GSH, and identified homozygous edited clones ( Figure 2B). These were named as follows: *AAVS1* T3 mCherry (AT3C_1/2/3/4/5; 5 clones) and *AAVS1* UCOE T3 mCherry (AUT3C_1/2/3/4/5/6; 6 clones). Table 2 show a summary of the targeting efficiency for this experiment. Of note, in this experiment most clones that underwent genotyping were pre-selected to show at least some degree of mCherry expression, as assessed by fluorescent microscopy following 24 h of dox treatment prior to clone picking for clonal expansion. This procedure revealed, unexpectedly, a larger number of colonies negative for mCherry, only a few of which were isolated to confirm whether they were correctly genome edited.

We analysed mCherry expression in the selected clones following dox treatment for 48 h ( Table 4; Figure 2C). Clones AT3C_1/2 and AUT3C_1/2/4/5 displayed homogeneous mCherry expression (>97%), while others had mixed mCherry+ and mCherry- populations. Two clones, ATC3_3 and AUT3C_3 showed <1% mCherry expression, which we subsequently confirmed to result from Tet-On 3G silencing. This variability is further illustrated by fluorescence microscopy images ( Figure 2D. mCherry MFI was slightly higher, though not significantly different, in UCOE-expressing clones at the pluripotent state ( Figure 2E). Overall, these findings indicated some degree of stochastic TRE3VG silencing at the hiPSC stage, with the UCOE sequence providing no clear protection against silencing and a marginal improvement of maximal promoter activity after dox treatment. Notably, no mCherry could be detected in dox-untreated cells also after the addition of UCOE ( Figure 2C), dissipating concerns regarding the possible leakiness induced by this modification. 

Given the unexpected results indicating frequent inefficiency of the OPTi-OX system in hiPSCs, we performed an experiment with the aim of quantifying this aspect more rigorously. To achieve this, we genome edited in parallel the WTC11 Tet-On 3G with each one of the four plasmids used up to this point, performing three technical replicates for each condition. After puromycin selection and cell expansion of a bulk population of all surviving cells (no monoclonal selection), we treated hiPSCs with dox for 48 h and quantified transgene expression *via* flow cytometry ( Figure 2F; Figure 2G). The results showed that an average of 7.25% of cells expressed mCherry when the TRE3VG promoter was alone, while 4.23% of cells were mCherry+ when TRE3VG was preceded by the UCOE sequence; this difference was, however, not statistically significant. In contrast, 27.6% of cells expressed EGFP with the TRE3VG promoter, while only 0.75% of cells with the T11 promoter showed any EGFP expression. The unexpectedly high rate of cells in which the OPTi-OX system was inactive can be explained by the subsequent finding that Tet-On 3G in the parental cell line used was frequently silenced. Despite this limitation, since all conditions were analysed in parallel, this experiment nevertheless allows us to confirm a stronger stability of the TRE3VG promoter compared to the T11 promoter. Moreover, the results suggest, unexpectedly, a greater silencing effect on the mCherry transgene compared to GFP. We speculate that the mCherry cDNA may be more susceptible to DNA methylation or other epigenetic modifications leading to silencing. Overall, these data confirm the possibility of silencing of the inducible promoters even in hiPSCs and provide a warning that this phenomenon is partially transgene sequence-dependent.

### Testing the Addition of UCOE to Enhance Inducible Transgene Expression in hiPSC-CMs

**Figure 3.**
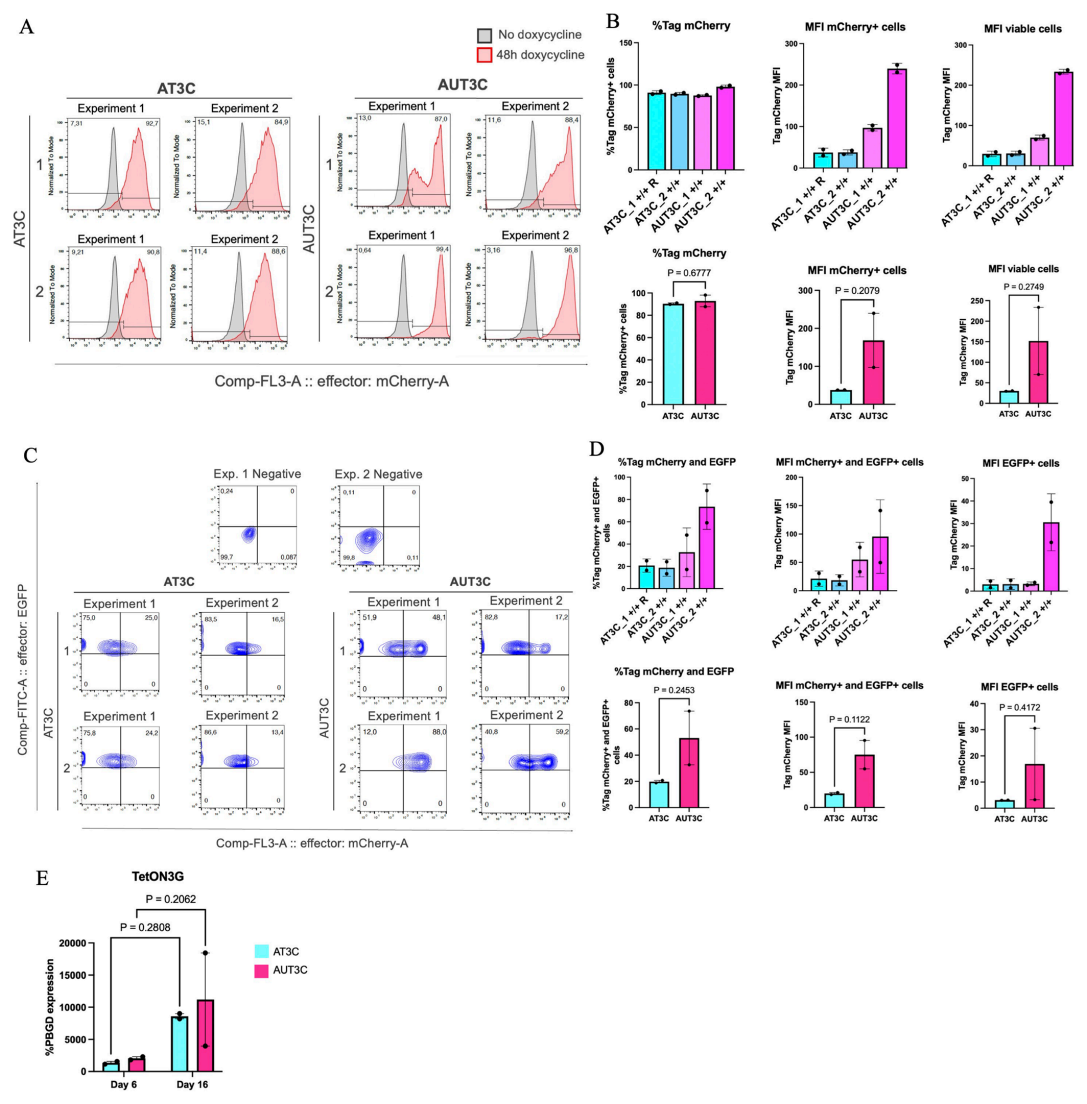
Testing the addition of UCOE to enhance inducible transgene expression in hiPSC-CMs. ( **A**) Flow cytometry analyses of mCherry expression in the four selected clones from Figure 2E at day 6 of cardiac differentiation. Cells were treated with dox for 48 h starting from day 4. Two biological replicates are reported. ( **B**) Flow cytometry quantifications in individual clones from panel A (top, n =2 biological replicates), and aggregate data for all clones from the same experimental condition (bottom, n = 2 clones). Statistical analyses by two-tailed t-test. ( **C**) Flow cytometry analyses as in panel A, but at day 16 of cardiac differentiation (cells dox-treated for 48 h from day 14). Biological replicates are reported. ( **D**) Flow cytometry quantifications in individual clones from panel C (top, n =2 biological replicates), and aggregate data for all clones from the same experimental condition (bottom, n = 2 clones). Statistical analyses by two-tailed t-test. ( **E**) RT-qPCR analysis of Tet-On 3G expression at day 6 and day 16 of cardiac differentiation. Statistical analysis by two-way ANOVA followed by post hoc Šidák multiple comparison test (n = 2 clones).

While UCOE did not appear to markedly improve inducible promoter expression in undifferentiated hiPSCs, we hypothesized that it could mitigate differentiation-associated promoter silencing. To evaluate this aspect during cardiac differentiation, we selected four *AAVS1*-edited clones with optimal activity of the OPTi-OX mCherry reporter, two with and two without UCOE (AUT3C_1/2 and AT3C_1/2, respectively). Cells were seeded at two different densities and differentiated using an established protocol based on biphasic modulation of the WNT signaling pathway ^28^. We analysed mCherry expression at day 6 (cardiac progenitors, CPs) and day 16 (CMs), repeating the experiment twice for biological replication. Cells were treated with dox for 48h prior to the collection time point. Figure 3 reports data for the seeding density (66 cells cm ^-2^) which led to more reproducible cardiac differentiation (average of 22.5% TTN+ at day 16).

At day 6 mCherry expression remained stable, with only a ~10% decrease in most clones, except for AUT3C_2, which maintained higher promoter activity ( Figure 3A). MFI was ~2–5 times higher in UCOE-expressing clones, a first hint that UCOE may reduce TRE3VG silencing during differentiation ( Figure 3B). These differences were, however, not statistically significant due to the substantial variation between the clones and their limited number. We also point out that as we did not stain for markers of CPs in this analysis (i.e., PDGFRA and CD56), we cannot demonstrate activity of the promoters specifically in CPs. Indeed, since the best seeding density resulted in only an a limited fraction of CMs, we can extrapolate that only a part of cells at this stage were CPs.

At day 16, mCherry expression was assessed only in TTN-GFP+ cells to specifically quantify promoter functionality in CMs ( Figure 3C). Marked silencing was observed in AT3C_1 and _2 clones, while UCOE-expressing clones maintained higher promoter activity, with an average of 32.65% and 7.6% of TTN+ cells still showing active promoter expression in AUT3C_1 and _2, respectively ( Figure 3D). While these results were not statistically significant for the same reasons mentioned above, the magnitude of the difference strongly suggest that the UCOE sequence can mitigate TRE3VG promoter silencing and maintain more stable transgene expression during cardiac differentiation.

To investigate the relationship between OPTi-OX activity and Tet-On 3G expression, we performed RT-qPCR for this transgene ( Figure 3E). Contrary to the trends in mCherry expression, Tet-On 3G increased an average of ~6 fold from day 6 to day 16; this rules out a major silencing issue of this transgene during differentiation, and suggests that reduced mCherry expression was indeed due to TRE3VG promoter silencing. Furthermore, results at day 16 demonstrate that there is not a substantial increase in Tet-On 3G expression in UCOE-expressing clones, meaning that the reduced mCherry expression in AT3C clones is indeed caused by promoter silencing and not by transactivator silencing.

### TRE3VG Promoter Activity at the *CLYBL* Locus in hiPSCs

**Figure 4.**
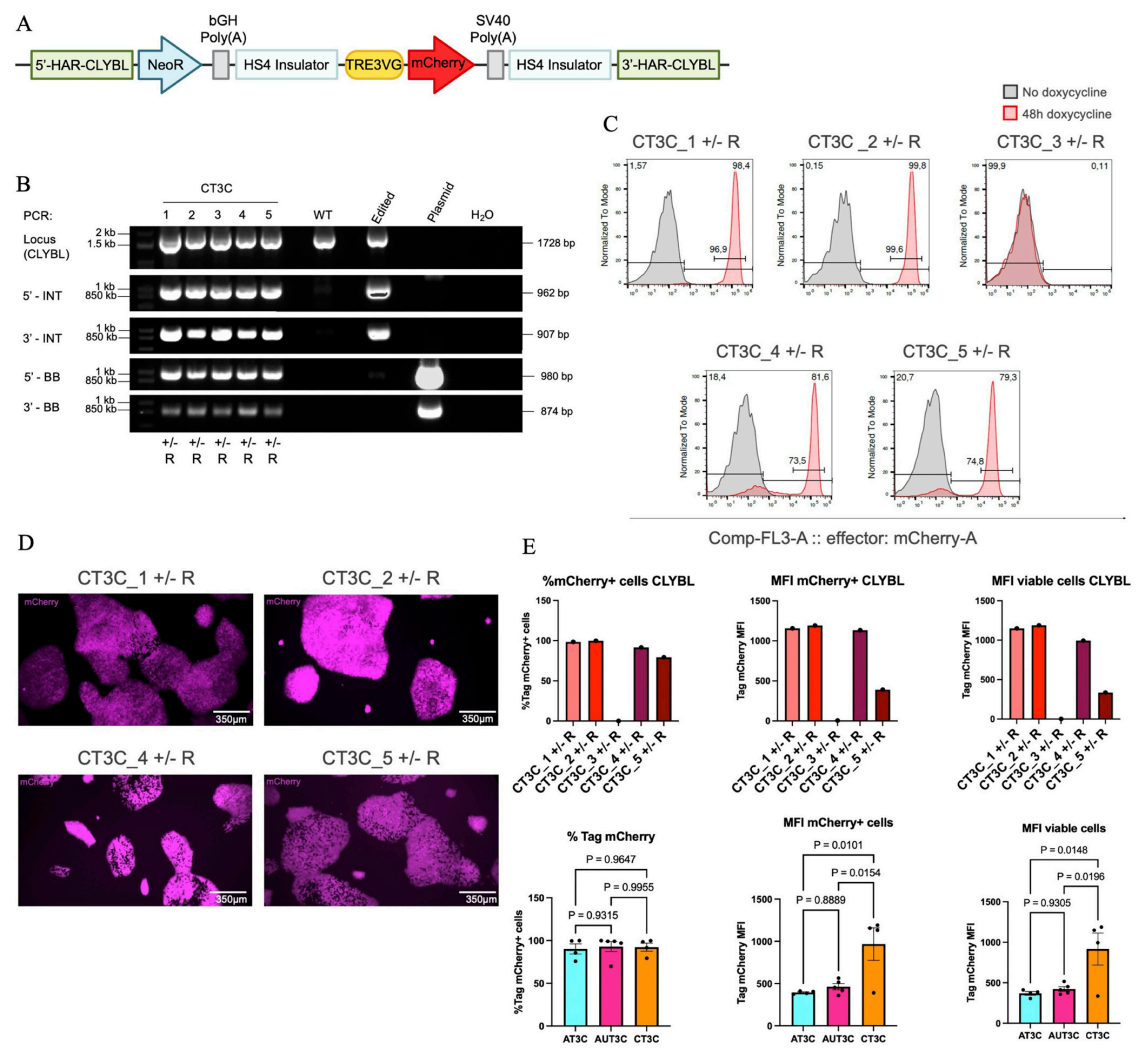
TRE3VG promoter activity at the *CLYBL* locus in hiPSCs. ( **A**) Schematic representation of the genome editing strategy. p *CLYBL*-Neo_TRE3VG_mCherry_Responder targets the *CLYBL* genomic locus and incorporates a neomycin resistance gene trap for selection (aminoglycoside-3'-phosphotransferase). HS4:chicken β-globin locus insulator. Refer to Figure 1A for other abbreviations. ( **B**) Genomic PCR verifying site-specific targeting of the *CLYBL* locus in WTC11 Tet-On 3G hiPSCs. Refer to Figure 1B for abbreviations. ( **C**– **D**) Flow cytometry analyses ( **C**) and fluorescence microscopy images of mCherry expression after treatment of hiPSC clones with dox for 48 h. In D, mCherry signal is shown in magenta; scale bars: 350 μm ( **E**) Flow cytometry quantifications in individual clones from panel C (top), and aggregate data for all clones compared to *AAVS1*-edited clones from Figure 2E (bottom). Statistical analyses by one-way ANOVA followed by post hoc Tukey’s multiple comparison. CT3C_1 and CT3C_2 clones were selected to proceed with cardiac differentiations.

To develop a strategy that prevents inducible promoter silencing during CM differentiation, we evaluated TRE3VG promoter activity at the *CLYBL* locus, hypothesizing it would support more stable and robust expression compared to the *AAVS1* locus. Since the *CLYBL* construct we used already contained insulator sequences ( Figure 4A), we did not include the UCOE. Indeed, the insulators are reported to protect against silencing due to formation of condensed chromatin through interfering with chromatin modifications ^29^. Besides the inducible cassette, the resulting plasmid contained a neomycin resistance gene trap. This construct was co-delivered with transcription activator-like effector nuclease (TALENs) selective for the intended insertion site into WTC11 Tet-On 3G hiPSCs *via* nucleofection. After neomycin selection and expansion of selected clones, genomic PCR confirmed site-specific targeting. Unlike *AAVS1* genome editing, which resulted in frequent homozygous targeting, all selected *CLYBL*-targeted clones ( *CLYBL* T3 mCherry: CT3C1/2/3/4/5; 5 clones) were heterozygous ( Figure 4B; Table 2).

Flow cytometry after 48h of dox treatment demonstrated a similar pattern to the *AAVS1*-targeted clones: some clones showed homogeneous mCherry expression, while others had mixed mCherry+ and mCherry- populations ( Figure 4C). Fluorescence microscopy images further illustrate variability in expression ( Figure 4D). One clone exhibited no mCherry expression due to lack of Tet-On 3G expression. On the other hand, MFI was ~1.9 fold higher in *CLYBL*-targeted clones than in both conditions of *AAVS1* targeting, with and without UCOE (p < 0.05 for both; Figure 4E; Table 4), indicating stronger activity of OPTi-OX based on the *CLYBL* GSH.

### TRE3VG Promoter Activity at the *CLYBL* Locus in hiPSC-CMs

To evaluate the functionality of TRE3GV in *CLYBL* during cardiac differentiation, we conducted further analyses on the two clones with the best OPTi-OX activity: CT3C_1 and CT3C_2. The experiment was conducted as for the clones targeting the *AAVS1* locus, testing two seeding densities to select the one leading to more robust differentiation (66 cells cm ^-2^), and analysing two different time points with two biological replicates.

**Figure 5.**
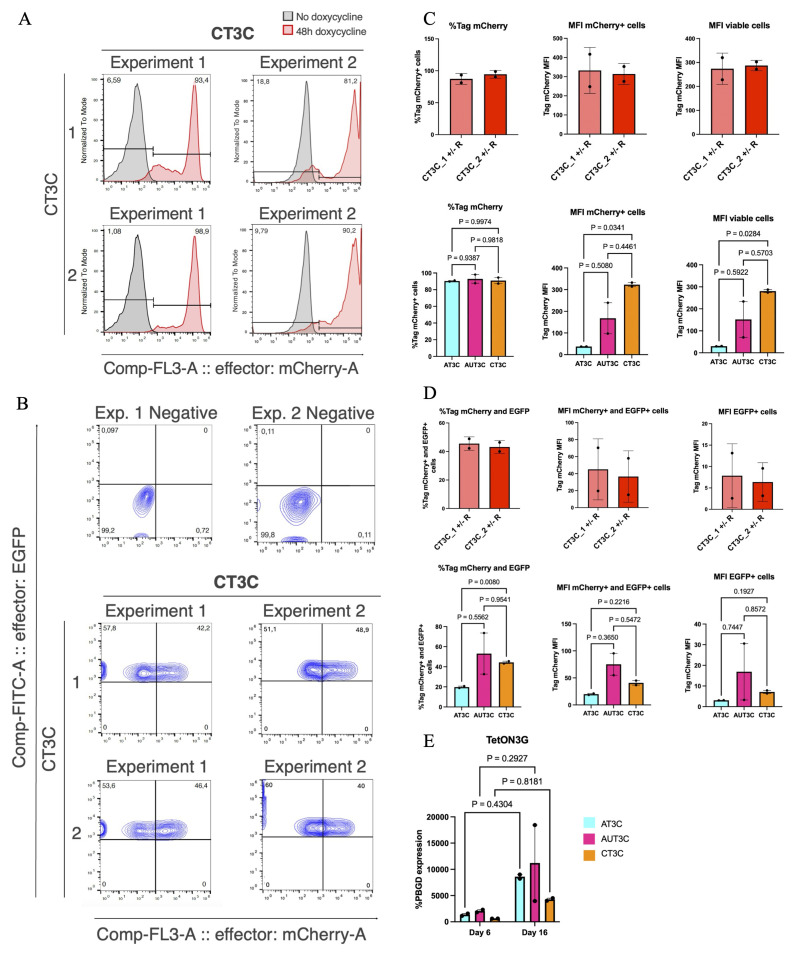
TRE3VG promoter activity at the *CLYBL* locus in hiPSC-CMs. ( **A**– **B**) Flow cytometry analysis of mCherry expression in the two selected clones from Figure 4E at day 6 ( **A**) or 16 ( **B**) of cardiac differentiation. Cells were treated with dox for 48 h starting from day 4 or day 14, respectively. Two biological replicates are reported. ( **C**– **D**) Flow cytometry quantifications in individual clones from panels **A**– **B**, respectively (top, n =2 biological replicates), and aggregate data for all clones compared to *AAVS1*-edited clones from Figure **B**, **D**, respectively (bottom, n = 2 clones). Statistical analyses by two-tailed one-way ANOVA followed by post hoc Dunnett’s T3 multiple comparison tests. ( **E**) RT-qPCR analysis of Tet-On 3G expression at day 6 and day 16 of cardiac differentiation, compared to the same analyses in *AAVS1*-edited clones from Figure 3E. Statistical analysis by repeated measures two-way ANOVA followed by post hoc Šidák multiple comparison test.

At day 6, similar to *AAVS1*-clones, mCherry expression remained relatively stable, with a decrease of only ~10% ( Figure 5A). At day 16, promoter activity was higher than the comparable *AAVS1*-targeted clones without the UCOE ( Figure 5B). Indeed, CT3C_1 and _2 clones respectively maintained 45.6% and 43.2% of mCherry expression at this later stage. This result was only marginally worse than the one observed in *AAVS1*-targeted clones with the UCOE. *CLYBL*-targeted clones exhibited MFI levels 2-fold higher to comparable *AAVS1*-targeted clones, and comparable to those observed in *AAVS1*-targeted clones carrying the UCOE-expressing clones ( Figure 5C and 5D). Analyses of Tet-On 3G expression by RT-qPCR confirmed upregulation of this transgene from day 6 to day 16, and comparable expression across various clone ( Figure 5E).

Collectively, these findings indicates that the *CLYBL* targeting strategy tested is preferable to the *AAVS1* one for OPTi-OX applications, though it is not sufficient to entirely prevent silencing of the TRE3VG promoter during cardiac differentiation.

### Sodium butyrate treatment to restore TRE3VG promoter activity in hiPSCs

**Figure 6.**
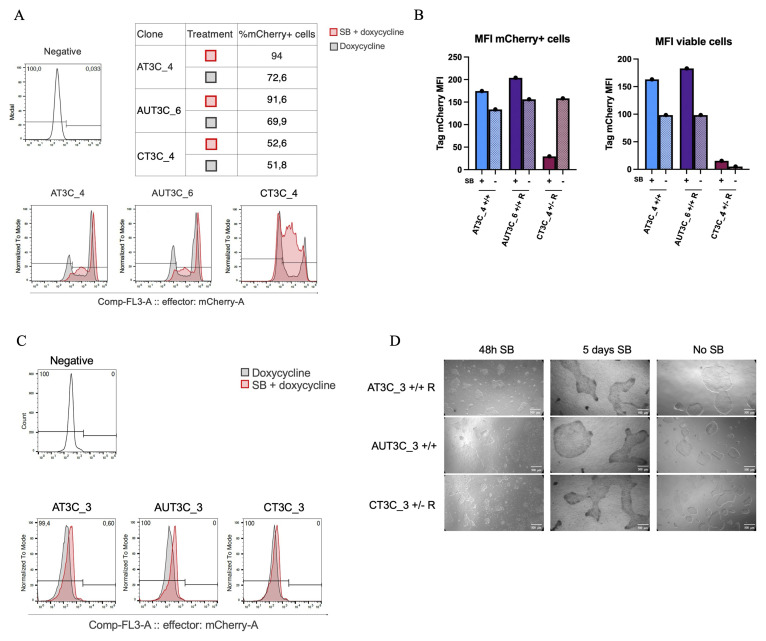
Sodium butyrate treatment to restore TRE3VG promoter activity in hiPSCs. ( **A**– **B**) Flow cytometry analyses ( **A**) and quantifications ( **B**) of mCherry expression in selected hiPSC clones with partial inactivity of the dox-inducible promoter. Cells were treated with either SB and dox or only dox for 72 h. ( **C**) Flow cytometry analyses of mCherry expression in selected hiPSC clones with complete inactivity of the dox-inducible promoter. Cells were treated with either SB and dox or only dox for 5 days. ( **D**) Phase contrast microscopy images of hiPSCs treated with SB and dox for 48 h and 5 days, compared to a dox-only 5 days control; scale bars = 500 μm.

To explore alternative methods to prevent and/or revert TRE3VG promoter silencing in hiPSCs and across cardiac differentiation, we tested the effectiveness of SB treatment, hypothesizing that it would enhance TRE3VG activity by promoting an open chromatin state. We began by analysing six hiPSC clones: AT3C_4, AUT3C _6, CT3C_4 (heterogeneous mCherry expression), and AT3C_3, AUT3C_3, CT3C_3 (no mCherry expression). The first three clones were treated with SB and dox for 3 days, followed by flow cytometry analysis ( Figure 6A). Except for CT3C_4, SB treatment increased mCherry expression by ~20% compared to controls treated only with dox, with a slight increase in MFI in the same two clones ( Figure 6B). For the completely silenced clones, we extended SB and dox treatment to 5 days, hypothesizing that longer treatment might help to restore promoter activity. Flow cytometry results ( Figure 6C) showed no mCherry expression restoration. These clones were later found to be lacking for Tet-On 3G expression, explaining why SB could not be effective in this context. Of note, despite the improvement in TRE3VG activity in partially silenced clones, SB negatively affected hiPSC morphology and viability ( Figure 6D).

### Sodium Butyrate treatment to Restore TRE3VG Promoter Activity in hiPSC-CMs

**Figure 7.**
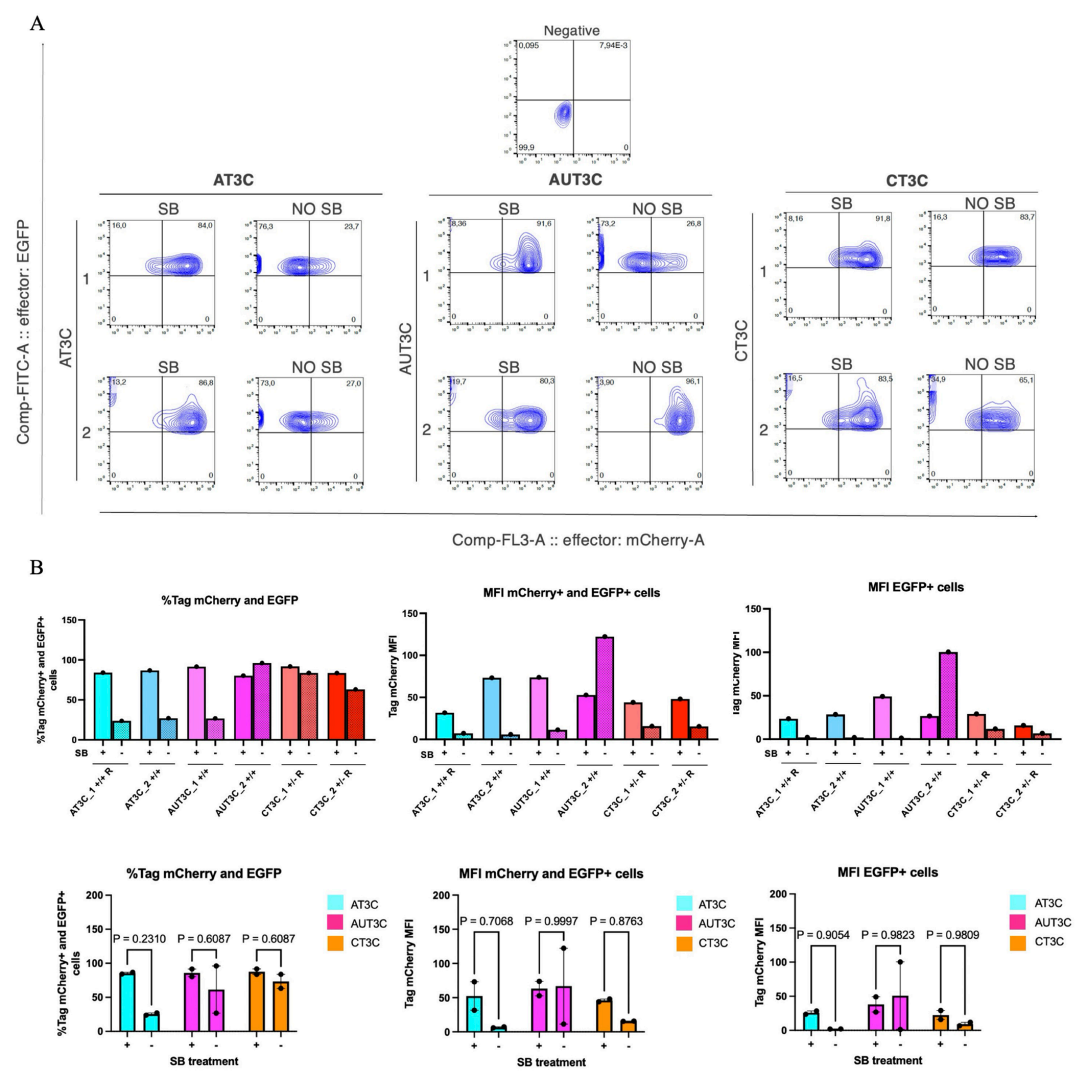
Sodium butyrate treatment to restore TRE3VG promoter activity in hiPSC-CMs. ( **A**) Flow cytometry analyses of mCherry expression in day 22 hiPSC-CMs from clones with full activity of the dox-inducible promoter in the iPSC stage. Cells were treated with SB and dox or only dox from day 17. ( **B**) Flow cytometry quantifications in individual clones from panel A (top), and aggregate data for all clones from the same experimental condition (bottom). Statistical analyses by repeated measures two-way ANOVA with post hoc Šidák multiple comparison test (n = 2 clones).

To test SB in hiPSC-CMs, we differentiated the two best clones from each condition (AT3C_1/2, AUT3C_1/2, CT3C _1/2) and analysed them by flow cytometry at day 22, after 5 days of treatment with SB and dox ( Figure 7A). SB treatment promoted OPTi-OX activity in all but one clone, and was particularly effective in clones with greater silencing (AT3C_1/2, AUT3C_1), increasing the fraction of mCherry expressing TTN+ cells by 30–50%—up to 80–90%—as well as improving MFI 4–12-fold ( Figure 7B). Cardiomyocyte morphology and viability were unaffected by SB. These results indicate that SB treatment can effectively restore TRE3VG promoter activity in CMs.

### Silencing of Tet-On 3G Transactivator

**Figure 8.**
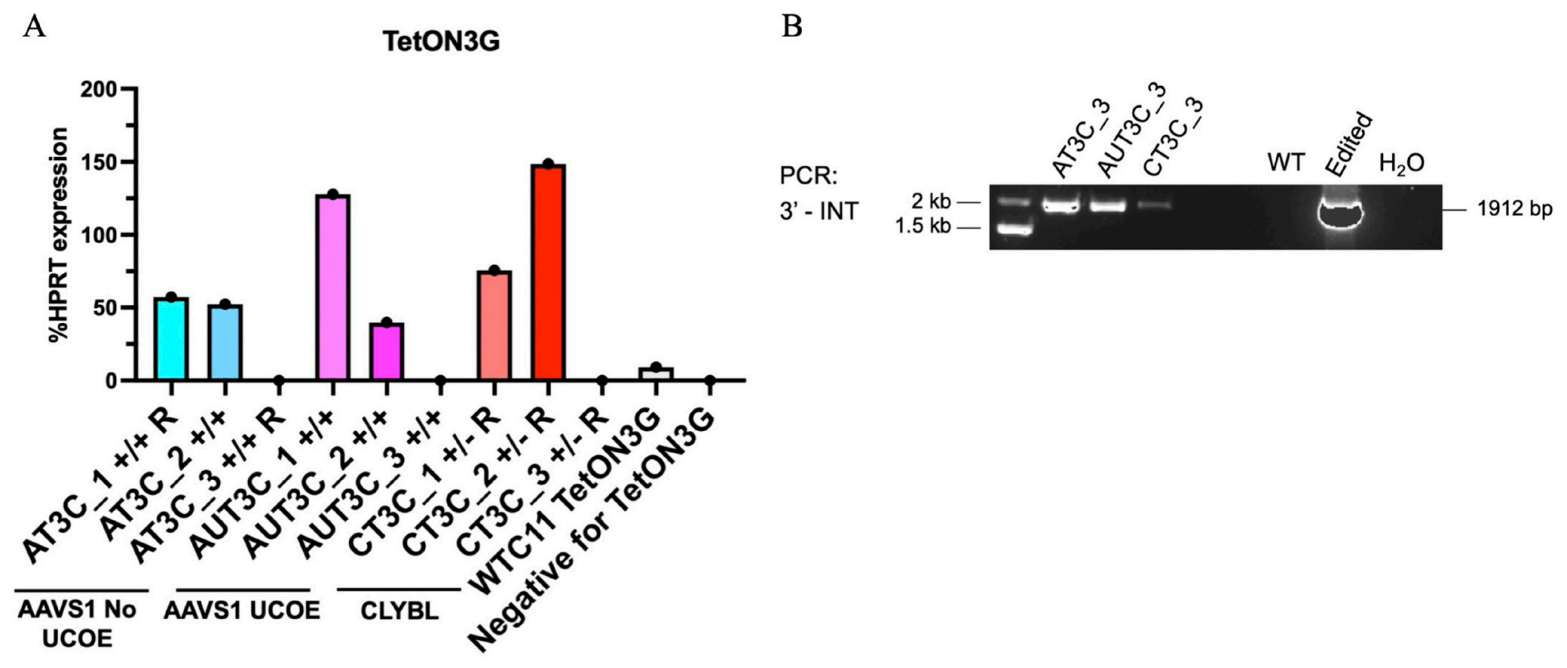
Silencing of Tet-On 3G transactivator. ( **A**) RT-qPCR analysis of Tet-On 3G expression in clones with full activity (#1 and #2) or complete inactivity (#3) of the dox-inducible promoters in the indicated genome editing strategies, compared to the parental WTC11 Tet-On 3G and TTN-GFP hiPSCs. ( **B**) Genomic PCR confirming the integration of the Tet-On 3G cassette in the clones with complete inactivity of the dox-inducible promoters. Refer to Figure 1B for abbreviations.

The ineffectiveness of SB treatment in restoring mCherry expression in fully silenced hiPSC clones (AT3C_3, AUT3C_3, and CT3C_3) prompted us to investigate alternative possibilities unrelated to the epigenetic silencing of the TRE3GV promoter. We considered the possibility that Tet-On 3G could be poorly expressed in these clones. To investigate this possibility, we performed RT-qPCR for Tet-On 3G in all selected clones with optimal OPTi-OX efficiency, (AT3C_1/2, AUT3C_1/2, CT3C _1/2), the three clones with dysfunctional OPTi-OX (AT3C_3, AUT3C_3, CT3C_3), and the parental WTC11 Tet-On 3G ( Figure 8A). Unexpectedly, Tet-On 3G was undetectable in all dysfunctional clones, and its expression was much lower in the WTC11 Tet-On 3G parental line compared to clones characterized by optimal activity of OPTi-OX. To rule out that this apparent silencing was merely the result of a genome editing error, we confirmed, through genomic PCR and Sanger sequencing, that the three clones with ineffective OPTi-OX had integrated the cassette expressing Tet-On 3G ( Figure 8B). Overall, these findings indicate that the parental line exhibited SB-refractory silencing of the transactivator, and suggests that the AT3C_3, AUT3C_3, and CT3C_3 clones likely originated from cells with pre-existing transactivator silencing. These observations also likely explain our unexpected results described in Figure 2F.

**Figure 9.**
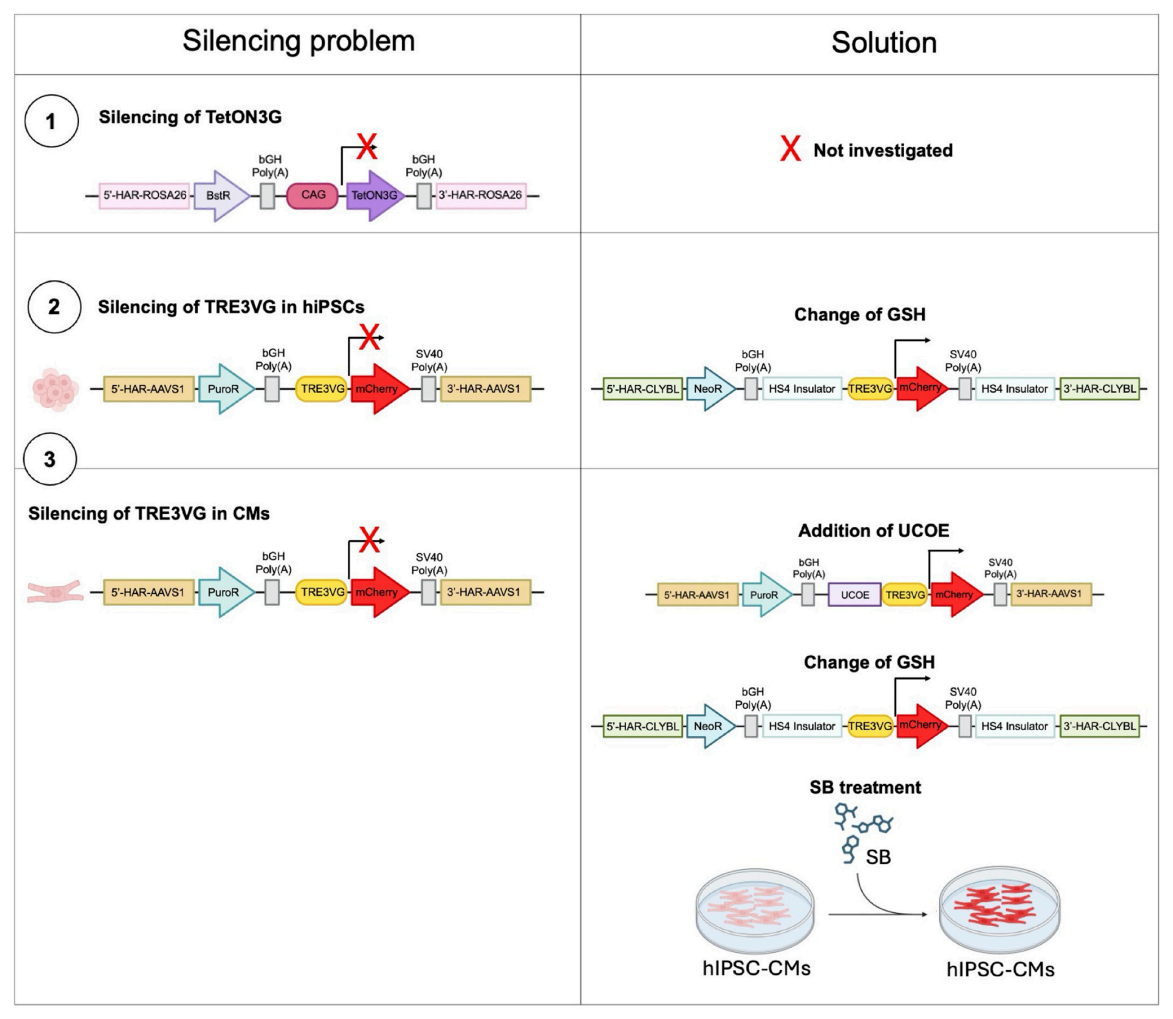
Working model. Summary of the mechanisms responsible for transgene expression silencing and the potential solutions explored to partially or fully restore transgene expression.

In conclusion, this study identified key mechanisms underlying transgene silencing in hiPSCs and hiPSC-CMs ( Figure 9). A major discovery was the irreversible silencing of the Tet-On 3G transactivator, which impeded expression in dysfunctional clones. Silencing of the TRE3VG promoter in hiPSCs was partially reduced by relocating it to the *CLYBL* locus. In CMs, both adding the UCOE at the *AAVS1* GSH and targeting the *CLYBL* GSH each modestly improved transgene expression, while SB treatment further enhanced promoter activity by promoting a more open chromatin state.

## Discussion

Our investigation focused on the challenge of ensuring stable, inducible transgene expression in hiPSCs and their differentiated progeny, specifically cardiomyocytes. Inducible promoters are critical for precise temporal and quantitative control of transgene expression, a key feature required in both basic and translational research. However, their tendency to undergo silencing during differentiation is a major obstacle, limiting their utility. Such silencing can be triggered by epigenetic modifications and the cellular environment, resulting in the loss of inducible transgene expression ^10^. The objective of this study was to identify strategies to prevent or reduce promoter silencing in hiPSCs and their hiPSC-CMs, specifically focusing on the cardiac lineage.

hiPSC-CMs generated through directed differentiation are often developmentally immature, which restricts their use in preclinical research and therapeutic applications ^30^. Indeed, in large animal models, while transplantation of hiPSC-CMs has been shown to restore cardiac function, it is also associated with significant risks, such as arrhythmias ^31– 34^. One strategy to enhance CMs maturity is through forward programming, where key cardiac transcription factors are overexpressed to drive the maturation process. Unfortunately, despite its potential, no established protocol for forward programming hiPSCs into fully mature CMs has been developed to date. We speculate that promoter silencing is one obstacle to this goal. Addressing this issue, therefore allowing the continuous expression of key transcription factors necessary for cardiac specification and maturation could facilitate the generation of more mature, adult-like CMs that are better suited for disease modelling, drug testing, and potential clinical applications. Consequently, by addressing promoter silencing, forward programming could offer a more direct and efficient path to generating CMs that are better suited for clinical applications. Beyond therapeutic applications, inducible promoters are invaluable for basic research, allowing scientists to control and modulate transgene expression in a quantitative and temporal way ^35^. Stable inducible systems are essential to obtain reproducible data and the study of gene function in various biological processes.

This study aimed to explore strategies to prevent or reduce silencing of these promoters, particularly in hiPSC-derived cardiac cells. Our findings indicate that the TRE3VG promoter is more robust than T11, but that silencing of TRE3VG remains a major issue during differentiation, limiting its utility in mature hiPSC-CMs. We tested several strategies to enhance the stability of TRE3VG expression, including the integration of a UCOE sequence and targeting alternative genomic loci. The UCOE did not significantly improve expression in undifferentiated hiPSCs, but showed modest benefits during differentiation. Additionally, integration of the TRE3VG-mCherry cassette into the *CLYBL* locus led to higher expression levels compared to the commonly used *AAVS1* locus, although some silencing still occurred during differentiation.

An important consideration is whether the observed differences in MFI and silencing between the *CLYBL* and *AAVS1* loci are due to the intrinsic properties of the *CLYBL* locus or the presence of insulators used in our construct. It is possible that the presence of these elements, rather than the genomic environment of the *CLYBL* locus itself, is contributing to the reduced silencing and enhanced expression observed. Either way, these results suggest that optimal genomic location alone may be insufficient to completely prevent promoter silencing, and interactions between promoter elements, chromatin modifiers, and specific loci need further investigation. Of note, however, we cannot rule out that it may be possible to identify a GSH exceptionally resistant to silencing among several alternatives reported in recent years ^36, 37^.

As a non-genetic alternative to address the issue of promoter silencing, we treated hiPSC-CMs with the epigenetic modulator SB, which was able to restore promoter activity. While the adverse effects of SB on hiPSC viability limit its use at the stem cell stage, this is not a major limitation since effective OPTi-OX hiPSCs can be readily isolated. On the other hand, application of SB in hiPSC-CMs did not have obvious effects on the cell morphology, though deeper analyses of transcriptional and functional changes should be performed to further establish the safety of this approach.

Surprisingly, our data indicates that Tet-On 3G transactivator silencing was a key roadblock in certain hiPSC clones. This is despite having introduced the transgene in a widely used GSH, *hROSA26*, and having used the strong constitutive promoter CAG, which we and others had previously shown to be strongly active in hiPSCs and its derivatives ^7, 38^. More recently, however, we found a similar issue when attempting to express a modified Cas9 protein using the same promoter from both the same locus as well as the *AAVS1* GSH ^7^, which confirms that CAG is not universally active in these loci and should be carefully tested.

Identifying the epigenetic modifications that lead to Tet-On 3G silencing, and developing strategies to maintain its activity, will be crucial for improving the establishment of OPTi-OX clones, particularly in the context of screening experiments. Notably, however, we did not observe silencing of Tet-On 3G in functional OPTi-OX clones during prolonged culture (up to 10 passages), which indicates that once isolated these can be safely used.

Future efforts should focus on optimizing the genomic environment of transgene constructs, using combinations of UCOE and other regulatory elements such as insulators, and exploring more specific epigenetic modifiers. One key area of focus will be the integration of the UCOE in combination with the *CLYBL* locus targeting strategy. Additionally, testing alternative dox-inducible promoters, such as the T8 promoter, could offer enhanced resistance to silencing due to unique transcription factor binding sites. Indeed, T8 consists of a wild-type MMTV promoter fragment that harbors binding sites for Oct-1 (FoxAI) and NF-1 ^13^. Moreover, understanding the mechanisms of promoter and transactivator silencing through techniques like methylation-specific PCR and chromatin immunoprecipitation will further enable the design of targeted interventions.

Overall, this study describes a number of strategies to overcome the complex issue of dox-inducible promoter silencing during hiPSC differentiation, and highlights that these may need to be combined in specific settings to achieve homogeneous inducible transgene expression. This provides an important step towards advancing hPSC-based models in both basic and translational research.

## Ethics and consent

Ethics and consent were not required.

## Data Availability

Zenodo: Underlying Data for Overcoming the Silencing of Doxycycline Inducible Promoters in hiPSC-derived Cardiomyocytes ^39^. DOI: https://doi.org/10.5281/zenodo.14163898 This project contains the following underlying data: Underlying Data 1.zip. Contains.tiff images underlying figures 1B, C and E and raw data .fcs from flow cytometry underlying figure 1D and F. Underlying Data 2.zip. Contains .tiff images underlying figures 2B, D and raw data .fcs from flow cytometry underlying figures 2C–G and 4C and E. Underlying Data 3.zip. Contains raw data .fcs from flow cytometry underlying figures 3A–D and 5A–D and raw data .eds from RT-qPCR underlying figure 3E and 5E. Underlying Data 4.zip. Contains .tiff images underlying figures 4B, D Underlying Data 5 .zip. Contains raw data .fcs from flow cytometry underlying figures 6A–C and .tiff images underlying figure 6D Underlying Data 6 .zip. Contains raw data .fcs from flow cytometry underlying figures 7A–B Underlying Data 7 .zip. Contains raw data .eds from RT-qPCR underlying figure 8A and .tiff image underlying figure 8B. Data are available under the terms of Creative Commons BY 4.0 licence.
