# Supplementary material for: Overcoming the Silencing of Doxycycline-Inducible Promoters in hiPSC-derived Cardiomyocytes
Source: Open Res Eur. 2024 Dec 18;4:266. [Version 1] doi: 10.12688/openreseurope.19024.1 (PMC11803382; doi:10.12688/openreseurope.19024.1)
Supplement: Peer review history

# Reviewer report: Reviewer response for version 1

**DOI:** 10.21956/openreseurope.20586.r48897

**Published:** 2025-02-06

**Authors:** Antonella Fidanza

**Affiliations:** 1 The University of Edinburgh, Edinburgh, Scotland, UK

Guichardaz et al. aim to address a major problem in genome editing, particularly the use of DOX-inducible systems in the hiPSC model. They apply and compare various strategies, providing interesting troubleshooting pathways. The manuscript is well-written, and the figures are well-presented.

 The method section is detailed and ensures reproducibility. It provides all the necessary information for other laboratories to repeat their experiments.

 The manuscript has excellent potential and impact; however, there are some crucial points for the authors to address to ensure their conclusions are robustly supported by the results.

**Main comments**

 It is crucial to deposit the plasmid to maximise the impact/reproducibility (I suggest Add gene). 

 The conclusion of TRE3 versus T11 activity is based on a single clone of TRE3 and then bulk comparison that, although more robust, might be affected by heterogenous integration events.

 The experiments have not all been repeated for n≥3; some data have stat with only n=2, which is incorrect. 

 The CLYBL to AAVS1 comparison might be affected by the different elements in the plasmids, namely insulators versus UCOE, and not necessarily the locus itself. 

**Minor comments**

 Include in the text that clone number 6 of ROSA26 was used later. 

 Why did they use clones with additional random integrations? The CLYBL clones all have additional integrations.

 Quantification of the viability after SB treatment is needed to support their conclusions. While data on morphology are provided, no data are available on viability. 

 Was the SB used throughout the CMs differentiation or only ahead of it? This needs to be clarified.  

 The naming of the underlying data does not match that of the data on the repository. Still, since the order is conserved it is easy to identify them, it might be better to rename for consistency.

Is the study design appropriate and does the work have academic merit?

Yes

Is the work clearly and accurately presented and does it cite the current literature?

Yes

If applicable, is the statistical analysis and its interpretation appropriate?

Partly

Are all the source data underlying the results available to ensure full reproducibility?

Yes

Are the conclusions drawn adequately supported by the results?

Partly

Are sufficient details of methods and analysis provided to allow replication by others?

Yes

Reviewer Expertise:

hiPSCs, genome editing, in vitro differentiation, transcriptomics, developmental biology.

I confirm that I have read this submission and believe that I have an appropriate level of expertise to confirm that it is of an acceptable scientific standard, however I have significant reservations, as outlined above.

# Reviewer report: Reviewer response for version 1

**DOI:** 10.21956/openreseurope.20586.r49845

**Published:** 2025-02-04

**Authors:** Sylwia Bobis-Wozowicz

**Affiliations:** 1 Jagiellonian University, Kraków, Poland

In their article, Guichardaz M, et al. investigated effective ways to overcome the silencing of inducible transgene expression in undifferentiated human induced pluripotent stem cells and during the differentiation of these cells into cardiomyocytes. For this purpose, they compared the activity of two inducible promoters, TR3VG and T11, integrated at the "safe harbor" locus, the *AAVS1* site.

 They showed that the TRE3VG promoter was more active than the T11 promoter. The Authors then tested whether insertion of the ubiquitous chromatin opening element (UCOE) upstream of the promoter would prevent transgene silencing.

 Unexpectedly, they found that UCOE didn't improve the persistence of transgene expression in a polyclonal iPSC population. However, UCOE improved transgene expression during cardiac differentiation of these cells. The Authors also analyzed the *CLYBL* "safe harbor" locus and concluded that it provides more stable transgene expression than the *AAVS1* locus.

 However, in this case, the transgenic cassette also contained two insulator sequences (HS4). Because of this difference, in my opinion, no definitive conclusions can be drawn about a stronger promoter activity at the *CLYBL* locus than at the *AAVS1* locus. The Authors should either test the two loci with the same transgenic cassette or modify the stated conclusions.

 It is only mentioned in the Discussion that the difference in promoter activity may be due to the presence of the insulators rather than the genomic context. This should already be explained in the Results section and Figure 9 should be modified accordingly.

 Importantly, all hiPSC clones targeted at the *CLYBL* locus also contained random integration events, which could be considered a drawback compared to the targeted integration efficiency at the *AAVS1* locus. This could also contribute to increased transgene expression (the number of random integration events was not evaluated) and should be discussed.

 Apart from these two major comments, the work is very solid, the methods are thoughtfully described in detail, allowing other researchers to reproduce the data. The results are presented clearly and the data analysis is performed accurately. The Authors provide useful solutions to overcome transgene silencing in two clinically relevant cell types. They present data on different promoter activities, transgenic cassette architecture, and the use of epigenome modifiers such as sodium butyrate. Thus, the results presented in this work may be useful for other researchers working with fluorescence-based molecular reporters.

Is the study design appropriate and does the work have academic merit?

Yes

Is the work clearly and accurately presented and does it cite the current literature?

Yes

If applicable, is the statistical analysis and its interpretation appropriate?

Yes

Are all the source data underlying the results available to ensure full reproducibility?

Yes

Are the conclusions drawn adequately supported by the results?

Partly

Are sufficient details of methods and analysis provided to allow replication by others?

Yes

Reviewer Expertise:

induced pluripotent stem cells, differentiation, cardiomyocytes, molecular reporters, "safe harbor", AAVS1 locus, single cell-derived clones, genome editing, designer nucleases, epigenetics.

I confirm that I have read this submission and believe that I have an appropriate level of expertise to confirm that it is of an acceptable scientific standard, however I have significant reservations, as outlined above.

# Reviewer report: Reviewer response for version 1

**DOI:** 10.21956/openreseurope.20586.r48893

**Published:** 2025-01-20

**Authors:** Vincenzo Cirulli

**Affiliations:** 1 University of Washington, Seattle, Washington, USA

In this study, Guichardaz and colleagues present an innovative combinatorial approach to address issues of transgene silencing often observed with gene editing experiments conducted in hiPSCs. Specifically, the authors focused on resolving previously noted gene silencing of doxycycline (dox)-inducible constructs targeted to genomic safe harbor sites such as *hROSA26* and *AAVS1. *Importantly, the study presents solid data from a series of experiments benchmarking strategies to enhance transgene expression, comparing two promoters (TRE3VG and T11), adding a chromatin opening element (UCOE), targeting the transgene cassette to an alternative genomic safe harbor site (CLYBL), and using sodium butyrate (SB) to reactivate silenced promoters.

 Key findings of the study include:

- TRE3VG showed higher activity than T11 but remained prone to silencing.
- UCOE modestly reduced silencing in hiPSC-CMs but did not boost activity in hiPSCs.
- Targeting the CLYBL locus enhanced promoter activity in both cell types.
- SB restored activity but reduced hiPSC viability and failed to reactivate silenced Tet-On 3G in some clones.

Collectively, these are important results that underscore the need for combining strategies like optimized genomic safe harbor site selection for specific cell type of interest, cassette design, epigenetic tools, and clone screening to improve the robustness of dox-inducible systems.

Is the study design appropriate and does the work have academic merit?

Yes

Is the work clearly and accurately presented and does it cite the current literature?

Yes

If applicable, is the statistical analysis and its interpretation appropriate?

Yes

Are all the source data underlying the results available to ensure full reproducibility?

Yes

Are the conclusions drawn adequately supported by the results?

Yes

Are sufficient details of methods and analysis provided to allow replication by others?

Yes

Reviewer Expertise:

Developmental biology. Gene editing of pluripotent stem cells.

I confirm that I have read this submission and believe that I have an appropriate level of expertise to confirm that it is of an acceptable scientific standard.
